# Association of leisure-time physical activity and resistance training with risk of incident hypertension: The Ansan and Ansung study of the Korean Genome and Epidemiology Study (KoGES)

**DOI:** 10.3389/fcvm.2023.1068852

**Published:** 2023-01-27

**Authors:** Jae Ho Park, Nam-Kyoo Lim, Hyun-Young Park

**Affiliations:** ^1^Division of Population Health Research, Department of Precision Medicine, Korea National Institute of Health, Korea Disease Control and Prevention Agency, Cheongju, Republic of Korea; ^2^Department of Precision Medicine, Korea National Institute of Health, Korea Disease Control and Prevention Agency, Cheongju, Republic of Korea

**Keywords:** hypertension, physical activity, resistance training, population study, prevention

## Abstract

Hypertension is the most common preventable risk factor for the onset of cardiovascular disease and mortality. We aimed to investigate the association between incident hypertension and 4-year leisure-time physical activity (PA) levels and resistance training (RT). In this community-based Korean cohort, 5,075 participants without hypertension were included. To evaluate cumulative PA, the average PA time (the total time of moderate-intensity leisure-time PA) at baseline, 2-year follow-up, and 4-year follow-up were calculated. Based on participation in RT and compliance to PA guidelines (≥150 min/week of PA time), the participants were divided into the following four groups: Low-PA, Low-PA+RT, High-PA, and High-PA+RT. A multivariate Cox proportional hazards regression model was used to evaluate the 12-year incidence of hypertension in relation to leisure-time PA levels and RT regularity. During a mean 7.86 ± 4.20-year follow-up, 2,544 participants (1,366 women) were diagnosed with hypertension. Compared with Low-PA, High-PA, and High-PA+RT decreased the risk for hypertension by 30 and 39%, respectively. Participation in RT without compliance to PA guidelines did not affect the incidence of hypertension. The additive effect of RT on hypertension in the High-PA group was further examined. Although sex-based comparisons indicated that men had a significantly longer training period for RT than women, an additional reduction in the risk for hypertension in relation to the addition of RT was observed only in women (35%). PA may confer protective effects against hypertension, whereas the addition of RT to high levels of PA can further reduce the risk for hypertension in women.

## 1. Introduction

High blood pressure (BP), clinically known as hypertension, is the most common preventable risk factor for cardiovascular disease (CVD)-related incidence and mortality, including those attributable to peripheral or coronary artery disease, heart failure, myocardial infarction, and stroke (1, 2). Worldwide, in 2019, the prevalence of hypertension was estimated at 1.3 billion, which indicated a twofold increase since 1990 (3). Therefore, hypertension is an important public health concern that imperatively necessitates the establishment of strategies to prevent hypertension to avoid life-threatening conditions.

The adoption and maintenance of lifestyle modifications, such as increased physical activity (PA), have been recommended for preventing and/or treating hypertension. To improve health-related physical fitness and prevent non-communicable diseases, including hypertension, diabetes mellitus, and CVD, the World Health Organization (WHO) has advised at least 150 min/week of moderate-intensity leisure-time PA (4). Epidemiological research revealed an inverse relationship between increased leisure-time PA levels and the risk for hypertension (5, 6). Randomized controlled trials (RCTs) have revealed that regular aerobic exercise training effectively decreases systolic BP (SBP) and diastolic BP (DBP) in patients who are diagnosed with resistant hypertension (7, 8). Taken together, participation in regular aerobic-related PA may confer protection against hypertension.

Unlike aerobic exercise training, which has a protective effect against hypertension, the association between resistance training (RT) and the risk of incident hypertension is controversial. RT–a type of PA characterized by muscle contraction against a force or weight–has been recommended for improving body composition, muscular fitness, BP levels, blood glucose levels, and insulin sensitivity (9–12). In RCTs with patients who had hypertension, moderate-intensity or progressive (from low- to moderate-intensity) RT was effective for decreasing both SBP and DBP (13, 14), whereas no changes in BP were noted following low-intensity isometric handgrip training (15); similarly, progressive (from low- to moderate-intensity) RT did not change BP in normotensive individuals (16). To the best of our knowledge, few prospective cohort studies have investigated the longitudinal association between RT and the risk for hypertension in initially normotensive participants. Recently, Mielke et al. (6) reported an inverse association between RT and a 6-year incidence of hypertension in an Australian cohort. However, as these authors did not consider clinical confounders that are related to incident hypertension, such as baseline BP, body mass index (BMI), and diabetes mellitus, further cohort studies are required to enhance the evidence of the association after adjusting for the known confounders. Moreover, data from other cohorts should be used to examine potential variations among races and ethnicities. There is a lack of studies investigating the combined associations of RT regularity and leisure-time PA levels with risk of incident hypertension.

Therefore, the present study was conducted to examine the association of hypertension with cumulative leisure-time PA levels and RT regularity in Korean adults from a population-based prospective cohort. Furthermore, we determined whether RT had an additive effect on hypertension in participants with high leisure-time PA levels.

## 2. Materials and methods

### 2.1. Study participants

The present study used data from the Korean Genome and Epidemiology Study (KoGES), conducted by the Korea National Institute of Health. The KoGES is a large consortium project that consists of six prospective cohort studies and that has been initiated with an aim to establish comprehensive healthcare guidelines for non-communicable diseases, such as obesity, metabolic syndrome, hypertension, diabetes mellitus, CVD, and cancer (17). In the present study, we used data from the KoGES Ansan and Ansung study, an ongoing prospective and population-based cohort study. As the amount of moderate-intensity leisure-time PA was measured from the third wave of the KoGES_Ansan and Ansung study, we performed the analysis considering these data as the baseline. Accordingly, this cohort included 7,515 participants aged 43–74 years who resided in Ansan (urban area) and Ansung (rural area) at baseline and were followed up biennially during a 12-year period, from the baseline (the third wave) in 2005–2006 to the ninth wave in 2017–2018. All participants underwent physical examinations and face-to-face surveys that were conducted by trained medical staff and a detailed description of these cohort studies was provided previously (17).

Among the 7,515 participants from the cohort, 2,440 participants were excluded from the present study based on the following exclusion criteria: lack of data on BP (*n* = 33), lack of data on PA levels (*n* = 529), no data available for the covariates (*n* = 86), and previously diagnosed with hypertension at baseline (*n* = 1,792). Thus, 5,075 participants (2,726 women) were included in the final analysis (Supplementary Figure 1). This study was approved by the Institutional Review Board Committee of the Korea National Institute of Health, Korea Disease Control and Prevention Agency (Approval No. 2021-04-02-P-A).

### 2.2. Measurement of PA

**FIGURE 1:**
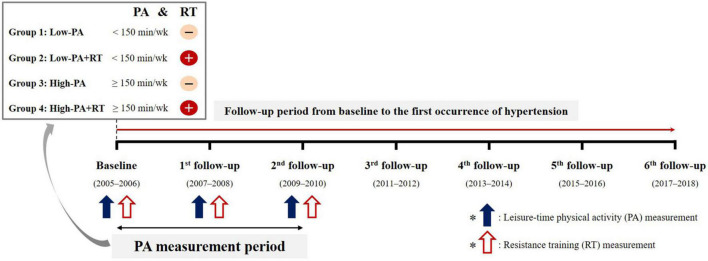
Scheme depicting the study grouping and follow-up over time.

Figure 1 shows the overall scheme of the study. All participants completed questionnaires to provide details of their regular leisure-time PA, including RT at baseline, 2-year follow-up, and 4-year follow-up. With regard to leisure-time PA, the frequency (per week), intensity, and duration (min) in a typical week were assessed. To evaluate the cumulative levels of PA over time, the average PA-time (total duration of moderate-intensity leisure-time PA; min/week) was calculated from the values reported at baseline, 2-year follow-up, and 4-year follow-up. Moderate-intensity leisure-time PA was considered as participation in exercise or sports to the point of sweating. However, to examine the cause-and-effect associations between PA levels and hypertension, if hypertension occurred during the PA measurement period, the average PA-time before the onset of hypertension was calculated. For example, in the case of participants who were diagnosed with hypertension at the 4-year follow-up, the average PA-time at baseline and 2-year follow-up was calculated. In the case of participants who were diagnosed with hypertension at the 2-year follow-up, the baseline PA-time was used in the analysis. Participants were classified into two groups in accordance with cutoffs specified on the basis of the WHO guideline of at least 150 min per week of moderate-intensity leisure-time PA as (4): “Low-PA” (not meeting the guideline) and “High-PA” (meeting the guideline).

RT was defined as any training program which involves muscle contraction against external resistance using body weight, weight machines, dumbbells, and barbells. For regularity of RT, the frequency (per week) and training period (months) of the most recent evaluation during the PA measurement period were assessed. However, to examine the cause-and-effect associations between RT and hypertension, if hypertension occurred during the PA measurement period, the most recent evaluation before the onset of hypertension was used. Regular RT was considered as participation in an RT program for more than 1 day per week. Participants were assigned to one of four subgroups based on RT regularity and leisure-time PA levels as follows: “Low-PA,” “Low-PA+RT,” “High-PA,” and “High-PA+RT.”

### 2.3. Definition of incident hypertension

Incident hypertension was defined as the first occurrence of hypertension in any follow-up period that was defined as SBP ≥ 140 mmHg, DBP ≥ 90 mmHg, treatment with antihypertensive drugs, or a diagnosis of hypertension by a physician (18). Trained healthcare providers measured BP using standard methods. SBP and DBP were considered as the average of two readings for the arm, with the highest SBP obtained after resting for 5 min while seated. For the comparison of baseline characteristics, the participants were divided into two groups based on incident hypertension during the follow-up period as follows: “Normotensive” (those without hypertension) and “Hypertension” (those with hypertension).

### 2.4. Covariates

Sociodemographic and health-related factors, including age, sex, drinking and smoking habits, education level, PA-time, BMI, waist circumference (WC), diabetes mellitus, and laboratory parameters, were included in our analyses. All covariates were derived from the baseline data, except the PA-time. Smoking and drinking habits were categorized as “never,” “former,” and “current.” The education level was divided into elementary school graduate or lower, middle or high school graduates, and college graduate or higher. PA-time was defined as the average value of the total time (min/week) of moderate-intensity leisure-time PA during the PA measurement period (baseline, 2-year follow-up, and 4-year follow-up).

Anthropometric data such as height, body weight, and WC were measured by trained healthcare providers using standardized protocols. BMI (kg/m^2^) was calculated as the body weight (kg) divided by height (m) squared. Blood samples were obtained after overnight fasting for at least 8 h. Furthermore, biochemical assays were performed to determine total cholesterol (T-Chol), high-density lipoprotein cholesterol (HDL-C), triglyceride (TG), fasting blood glucose (FBG), and creatinine levels. The estimated glomerular filtration rate (eGFR) was calculated using the following formula, with creatinine expressed in mg/dL (19): eGFR (ml/min per 1.73 m^2^) = 175 × (creatinine)^–1.154^ × (age)^–0.203^ × (0.742, if female). Diabetes mellitus was defined based on a previous diagnosis by a physician, current use of anti-diabetes medications, including insulin and oral anti-diabetes agents, FBG ≥ 126 mg/dL, or glycated hemoglobin ≥ 6.5. A detailed description of the biochemical analysis is available elsewhere (17).

### 2.5. Statistical analysis

All statistical analyses were conducted using SAS (version 9.4; SAS Institute, Cary, North Carolina, United States). The baseline characteristics of the participants are presented as descriptive statistics. Continuous variables are shown as mean ± standard deviation, whereas categorical variables are expressed as absolute frequency and percentages (%). The chi-square test was used to determine intergroup differences in sex ratios, drinking and smoking habits, education levels, participation in RT, and prevalence of diabetes mellitus. *Post-hoc* pairwise comparisons were used if significant differences were found when there were three or more levels of the categorical variables. Independent *t*-tests were used to compare age, PA-time, BMI, WC, SBP, DBP, T-Chol, HDL-C, TG, FBG, creatinine, and eGFR between the groups. The folded *F*-test was used to assess assumptions of equal variances. The pooled *t*-test was used when the variance was homogeneous between the two groups, whereas the Satterthwaite *t*-test was used when the variance was assumed unequal.

A multivariate Cox proportional hazards regression model was used to evaluate the hazard ratios (HRs) and 95% confidence intervals (CIs) for the incidence of hypertension. The models were adjusted for age, sex, drinking, smoking, education level, BMI, T-Chol, SBP, eGFR, PA-time, and diabetes mellitus. Kaplan–Meier curves and log-rank tests were used to compare the cumulative risk of hypertension among the groups. We performed a subgroup analysis of the association between leisure-time PA levels and incident hypertension according to age (<55 and ≥55 years), sex (male and female), BMI (<25 and ≥25 kg/m^2^), current drinking habits (no and yes), smoking status (never or ever), and diabetes mellitus (no and yes). The *p*-value for the interaction was estimated to investigate the consistency of the patterns of associations across subgroups. All tests were two-tailed, and statistical significance was set at a *p* < 0.05.

## 3. Results

A total of 5,075 participants (2,726 women) were enrolled in the present study. The mean follow-up period was 7.86 ± 4.20 years (range 1.33–12.59 years). During the follow-up period, 2,544 participants (50.1%) developed hypertension. Table 1 shows the baseline characteristics of the participants based on their leisure-time PA levels and sex. In both sexes, the mean age, SBP, and DBP were significantly lower in the High-PA group compared with the Low-PA group. The prevalence of current drinking was lower only in women in the Low-PA group, whereas the eGFR and the proportion of current smokers were higher only in men in the Low-PA group. Both men and women in the Low-PA group showed a considerably higher prevalence of low educational levels (≤elementary school) than that in those in the High-PA group. In men, compared to the Low-PA group, the High-PA group was significantly associated with higher regularity of RT, BMI, T-Chol, creatinine, and prevalence of diabetes mellitus. In women, compared to the Low-PA group, the High-PA group had significantly higher regularity of RT, T-Chol, HDL-C, and creatinine, but lower WC, TG, and FBG.

**TABLE 1:** Baseline characteristics of study participants stratified by leisure-time physical activity (PA) levels.

Variables	Men (*n* = 2,349)		*p*-value	Women (*n* = 2,726)		*p*-value
	Low-PA (*n* = 1,401)	High-PA (*n* = 948)		Low-PA (*n* = 1,744)	High-PA (*n* = 982)	
Age (years)	56.32 ± 8.80	53.54 ± 7.90	<0.0001	56.81 ± 8.99	52.84 ± 7.58	<0.0001
Education level, *n* (%)			<0.0001			<0.0001
≤ Elementary school	355 (25.34)	87 (9.18)^a^		864 (49.54)	266 (27.09)^a^	
Middle/high school	897 (64.03)	641 (67.61)^b^		818 (46.90)	654 (66.60)^b^	
≥ College	149 (10.63)	220 (23.21)^c^		62 (3.56)	62 (6.31)^b^	
Drinking habit, *n* (%)			0.69			<0.001
Never drinker	287 (20.49)	193 (20.36)		1,307 (74.94)	662 (67.41)^a^	
Ex-drinker	134 (9.56)	81 (8.54)		26 (1.49)	15 (1.53)^a,b^	
Current drinker	980 (69.95)	674 (71.10)		411 (23.57)	305 (31.06)^b^	
Smoking habit, *n* (%)			<0.0001			0.32
Never smoker	337 (24.05)	250 (26.37)^a^		1,694 (97.13)	956 (97.35)	
Ex-smoker	475 (33.91)	401 (42.30)^a^		8 (0.46)	8 (0.81)	
Current smoker	589 (42.04)	297 (31.33)^b^		42 (2.41)	18 (1.84)	
PA-time (min/week)	34.39 ± 50.52	334.72 ± 170.85	<0.0001	37.93 ± 52.42	318.69 ± 159.03	<0.0001
RT, *n* (%)	56 (4.00)	226 (23.84)	<0.0001	37 (2.12)	185 (18.84)	<0.0001
BMI (kg/m^2^)	23.64 ± 2.99	24.25 ± 2.53	<0.0001	24.49 ± 3.17	24.57 ± 2.92	0.53
WC (cm)	84.45 ± 8.09	84.07 ± 7.13	0.24	84.22 ± 9.58	80.87 ± 8.92	<0.0001
SBP (mmHg)	112.76 ± 11.49	110.79 ± 10.85	<0.0001	111.77 ± 12.76	108.13 ± 12.55	<0.0001
DBP (mmHg)	76.23 ± 7.43	75.03 ± 7.42	<0.001	74.12 ± 8.18	72.14 ± 8.30	<0.0001
T-Chol (mg/dL)	183.76 ± 33.48	190.15 ± 32.59	<0.0001	192.90 ± 34.23	195.66 ± 33.46	<0.05
HDL-C (mg/dL)	42.49 ± 10.33	43.00 ± 10.06	0.23	44.91 ± 9.65	46.80 ± 10.58	<0.0001
TG (mg/dL)	146.74 ± 121.91	144.80 ± 95.69	0.67	127.59 ± 80.24	117.31 ± 66.43	<0.001
FBG (mg/dL)	93.02 ± 13.42	94.15 ± 16.05	0.09	89.91 ± 12.80	88.35 ± 11.21	<0.01
Creatinine (mg/dL)	1.05 ± 0.13	1.09 ± 0.13	<0.0001	0.87 ± 0.11	0.89 ± 0.24	<0.05
eGFR (ml/min per 1.73 m^2^)	74.49 ± 11.11	72.22 ± 9.66	<0.0001	68.27 ± 9.49	67.80 ± 8.56	0.19
DM, *n* (%)	134 (9.56)	115 (12.13)	<0.05	164 (9.40)	74 (7.54)	0.10

PA, physical activity; PA-time, total time spent for regular participation in any sport or exercise to the point of sweating; min, minute; RT, resistance training; BMI, body mass index; WC, waist circumference; SBP, systolic blood pressure; DBP, diastolic blood pressure; T-Chol, total cholesterol; HDL-C, high-density lipoprotein cholesterol; TG, triglycerides; FBG, fasting blood glucose; eGFR, estimated glomerular filtration rate; DM, diabetes mellitus. Categories marked with the same letter are not significantly different by *post-hoc* pairwise comparisons.

The baseline characteristics of the participants, stratified based on incident hypertension and sex during the follow-up period, are shown in Supplementary Table 1. The incidence rates for hypertension in our study population were similar in both sexes (50.15 and 50.11% in men and women, respectively). Importantly, compared to the Normotensive group, the Hypertension group had significantly lower PA-time and proportion of regularity of RT. The mean age, BMI, and WC were significantly higher in the Hypertension group than in the Normotensive group. Furthermore, the prevalence of low educational level (≤elementary school) was higher in the Hypertension group in both sexes, whereas the proportion of current drinking was lower only in women in the Hypertension group. In both sexes, compared to the Normotensive group, the Hypertension group had significantly higher SBP, DBP, TG, FBG, and prevalence of diabetes mellitus, but had lower HDL-C.

Table 2 presents the inverse association between leisure-time PA levels and incidence of hypertension after adjusting for covariates. High levels of leisure-time PA were related to a decrease in the risk of hypertension in 31 and 35% of men and women, respectively (all *p* < 0.0001).

**TABLE 2:** Leisure-time physical activity (PA) level- and sex-stratified hazard ratios for incident hypertension.

	*N*	PA-time (min/week)	Total person-years	Participants with hypertension, *n* (%)	Event rate (1,000-person year)	Crude model	Model 1	Model 2
						HR (95% CI)	HR (95% CI)	HR (95% CI)
**Total**								
Low-PA	3,145	36.35 ± 51.61	20,894.85	1,776 (56.47)	85.00	1 (reference)	1 (reference)	1 (reference)
High-PA	1,930	326.56 ± 165.09	16,002.79	768 (39.79)	47.99	0.58 (0.53–0.64)****	0.70 (0.64–0.77)****	0.68 (0.62–0.74)****
**Men**								
Low-PA	1,401	34.39 ± 50.52	9,135.43	788 (56.25)	86.26	1 (reference)	1 (reference)	1 (reference)
High-PA	948	334.72 ± 170.85	7,659.42	390 (41.14)	50.92	0.64 (0.56–0.73)****	0.71 (0.62–0.81)****	0.69 (0.60–0.79)****
**Women**								
Low-PA	1,744	37.93 ± 52.42	11,759.42	988 (56.65)	84.02	1 (reference)	1 (reference)	1 (reference)
High-PA	982	318.69 ± 159.03	8,343.37	378 (38.49)	45.31	0.53 (0.47–0.60)****	0.70 (0.62–0.81)****	0.65 (0.57–0.75)****

PA, physical activity; PA-time, time spent for regular participation in any sport or exercise to the point of sweating; HR, hazard ratio; CI, confidence interval; BMI, body mass index; T-Chol, total cholesterol; SBP, systolic blood pressure; eGFR, estimated glomerular filtration rate.

****p < 0.0001.

Model 1 was adjusted for age, sex, drinking, smoking, and education level.

Model 2 was adjusted for the variables in Model 1, in addition to BMI, T-Chol, SBP, eGFR, and diabetes mellitus.

**FIGURE 2:**
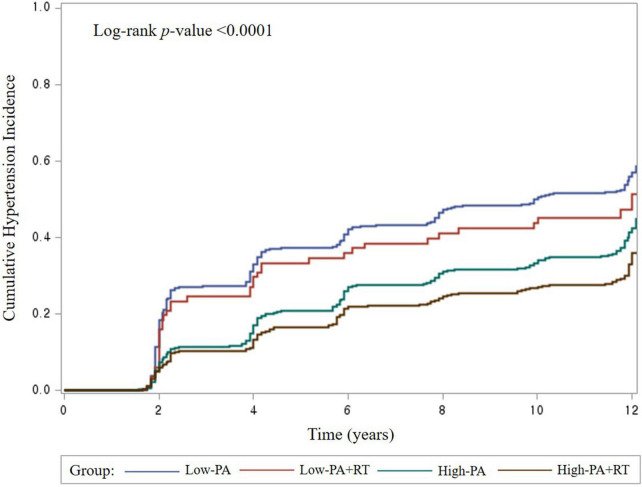
Cumulative incidence of hypertension in the four subgroups.

The additional effect of RT on hypertension among participants with low or high leisure-time PA levels was further investigated. The proportion of male and female participants who engaged in RT was 12.01 and 8.14%, respectively. The participants were divided into four subgroups based on leisure-time PA levels and RT. As shown in Figure 2, the Low-PA group exhibited the highest cumulative incidence rate of hypertension (log-rank *p* < 0.0001). RT showed no significant impact in the Low-PA group with regard to hypertension (Table 3). However, compared to the Low-PA group, the High-PA, and High-PA+RT groups had 30 and 39% lower risks for hypertension, respectively (all *p* < 0.0001; *p* for trend <0.0001). These associations remained significant, even after stratifying the participants by sex. Among men, the High-PA+RT group had a markedly higher training frequency (*p* < 0.0001) and a higher rate of long-term RT program (≥1 year; *p* < 0.01) than the Low-PA+RT group. Among women, the High-PA+RT group had a significantly higher training frequency (*p* < 0.01), a longer RT period (*p* < 0.05), and a higher rate of long-term RT program (≥1 year; *p* < 0.0001) than the Low-PA+RT group. The baseline characteristics of the four subgroups are shown in Supplementary Table 2.

**TABLE 3:** Leisure-time physical activity (PA) level-, resistance training (RT) regularity-, and sex-stratified hazard ratios for incident hypertension.

	*N*	Total person-years	Participants with hypertension, *n* (%)	Event rate, (1,000-person year)	PA-time, (min/week)	RT Levels			Crude model, HR (95% CI)	Adjusted model, HR (95% CI)
						Frequency	Training period			
						(days/week)	(month)	≥1 year (%)		
**Total**										
Low-PA	3,052	20,255.66	1,726 (56.55)	85.21	34.69 ± 50.94	–	–	–	1 (reference)^a^	1 (reference)^a^
Low-PA+RT	93	639.19	50 (53.76)	78.22	90.74 ± 43.11	3.72 ± 1.68^d^	59.69 ± 110.81	49.46^d^	0.85 (0.62–1.17)	1.13 (0.82–1.56)
High-PA	1,519	12,358.07	629 (41.41)	50.90	312.30 ± 158.39	–	–	–	0.61 (0.55–0.67)****	0.70 (0.63–0.77)****
High-PA+RT	411	3,644.72	139 (33.82)	38.14	379.28 ± 178.36	4.69 ± 1.48^d^	67.12 ± 81.23	74.21^d^	0.48 (0.40–0.57)****	0.61 (0.51–0.74)****
**Men**										
Low-PA	1,345	8,800.11	756 (56.21)	85.91	32.53 ± 49.98	–	–	–	1 (reference)^a^	1 (reference)^a^
Low-PA+RT	56	335.32	32 (57.14)	95.43	79.12 ± 42.61	3.49 ± 1.75^d^	87.54 ± 137.10	60.71^c^	1.02 (0.68–1.53)	1.29 (0.85–1.97)
High-PA	722	5,747.93	307 (42.52)	53.41	321.62 ± 162.71	–	–	–	0.66 (0.58–0.77)****	0.71 (0.61–0.83)****
High-PA+RT	226	1,911.49	83 (36.73)	43.42	376.58 ± 188.96	4.66 ± 1.62^d^	85.47 ± 96.78	77.88^c^	0.56 (0.44–0.71)****	0.66 (0.51–0.84)**
**Women**										
Low-PA	1,707	11,455.55	970 (56.82)	84.68	36.40 ± 51.65	–	–	–	1 (reference)^a^	1 (reference)^a^
Low-PA+RT	37	303.87	18 (48.65)	59.24	108.32 ± 38.03	4.00 ± 1.56^c^	25.09 ± 47.82^b^	32.43^d^	0.69 (0.41–1.15)	0.97 (0.58–1.63)
High-PA	797	6,610.14	322 (40.40)	48.71	303.86 ± 153.99	–	–	–	0.56 (0.49–0.65)****	0.67 (0.58–0.77)****
High-PA+RT	185	1,733.23	56 (30.27)	32.31	382.59 ± 164.95	4.72 ± 1.32 ^c^	45.80 ± 50.66 ^b^	69.73 ^d^	0.39 (0.29–0.52)****	0.56 (0.42–0.76)***

PA, physical activity; RT, resistance training; PA-time, total time spent for regular participation in any sport or exercise to the point of sweating; HR, hazard ratio; CI, confidence interval; BMI, body mass index; T-Chol, total cholesterol; SBP, systolic blood pressure; eGFR, estimated glomerular filtration rate.

^a^*p* < 0.0001 in the test for trend of HRs.

^b^*p* < 0.05 compared Low-PA+RT with High-PA+RT.

^c^*p* < 0.01 compared Low-PA+RT with High-PA+RT.

^d^*p* < 0.0001 compared Low-PA+RT with High-PA+RT.

**p < 0.01; ***p < 0.001; ****p < 0.0001; Adjusted for age, sex, drinking, smoking, education level, BMI, T-Chol, SBP, eGFR, and diabetes mellitus.

We analyzed the additive effect of RT on hypertension in the High-PA group (Supplementary Table 3). Interestingly, although the sex-stratified comparisons indicated that men had a considerably longer training period for RT than women (*p* < 0.0001), further reduction in the risk of hypertension related to the addition of RT was seen only in women (HR, 0.65; 95% CI, 0.46–0.91; *p* < 0.05).

Subgroup analysis was performed based on age, sex, BMI, current drinking habits, smoking status, and the diabetes mellitus (Supplementary Table 4). The protective benefit of high leisure-time PA levels against hypertension was consistent across all subgroups in the analyses based on age, sex, BMI, current drinking habits, smoking status, and diabetes mellitus. A significant interaction was observed for current drinking habits (no and yes; *p* for interaction <0.01); however, in both subgroups, high leisure-time PA conferred a protective benefit against hypertension.

## 4. Discussion

The present study indicates that satisfying the leisure-time PA guideline (≥150 min/week of moderate-intensity PA) may be associated with protective benefits against hypertension, whereas the addition of RT to high levels of PA seemed to be relatively more effective in preventing hypertension. In the subgroup analysis that was conducted to examine whether RT had an additive effect on the risk reduction for hypertension in the High-PA group, further decrease in risk related to the addition of RT was seen only in women. To the best of our knowledge, this is the first study to examine the longitudinal associations of hypertension with cumulative leisure-time PA levels and RT regularity in Korean adults using population-based data from a large cohort.

The adoption of lifestyle modifications is recommended to prevent and/or treat hypertension. In particular, current guidelines recommend at least 150 min per week of moderate-intensity leisure-time PA or sports to prevent and/or treat hypertension (4, 10). It is well known that high levels of leisure-time PA levels are inversely associated with hypertension. The majority of studies on hypertension have focused on the effect of regular aerobic-related leisure-time PA. Several epidemiological studies have reported an inverse longitudinal association between increased moderate-to-vigorous intensity leisure-time PA levels and the risk for hypertension (5, 6, 20, 21). In our study, compliance with the leisure-time PA guidelines was associated with a 32% reduction in the risk of incident hypertension when compared with PA levels below the current recommendation, after adjusting for other confounders. Moreover, this protective association persisted even after subgroup analysis performed by age, sex, BMI, current drinking habit, smoking status, and diabetes mellitus. Our results are consistent with those of previous studies. A meta-analysis of cohort studies revealed a linear dose–response association between leisure-time PA and incident hypertension, even after adjusting for BMI, and this indicated that the protective effect on hypertension increased with an increased amount of PA (22). Potential mechanisms for the reduction in BP after regular aerobic-related leisure-time PA include an improvement in vascular endothelial function based on increased nitric oxide (NO) bioavailability (23) and a reduction in microvascular rarefaction through enhanced endothelial progenitor cell-mediated angiogenesis (24). Taken together, our findings and those of previous studies suggest that increasing leisure-time PA levels is likely to be effective for mitigating the risk of incident hypertension.

Unlike aerobic-related leisure-time PA, which has a protective effect on hypertension, the association between muscular strength or muscle-strengthening PA, such as RT, and the risk for hypertension is controversial. In a recent cross-sectional study, we found that higher levels of handgrip strength were related to a reduction in the risk for hypertension (25). However, considering the nature of this cross-sectional study, further research is required. Recently, a prospective cohort study showed that participation in RT was related to a lower risk for hypertension in an Australian cohort, although baseline BP, BMI, leisure-time PA levels, and diabetes mellitus were not considered as confounders that are related to hypertension (6). Accordingly, we further investigated the longitudinal associations of hypertension with cumulative leisure-time PA levels and RT regularity in Korean adults. The present study indicates that RT had no effect on the risk for hypertension in the Low-PA group. Both the rate of participation in the long-term RT program (≥1 year) and RT frequency were significantly higher in the High-PA group than in the Low-PA group for both sexes; however, information on the training intensity was unavailable. Compared with the Low-PA group, high levels of leisure-time PA reduced the risk of incident hypertension by 30%, and the addition of RT to high levels of leisure-time PA further reduced the risk by 39% even after adjusting for covariates. However, the subgroup analysis of participants with high leisure-time PA levels revealed that RT further decreased the incidence of hypertension, particularly in women. This is consistent with the findings of recent meta-analyses of RCTs, which reported that moderate-intensity RT significantly reduced SBP and DBP in pre-hypertensive and hypertensive individuals (26, 27). However, several RCTs have indicated the lack of change in BP following progressive (from low- to moderate-intensity) RT (16), low-intensity isometric handgrip training (15), and high-intensity RT (28, 29). Thus, the antihypertensive effects of RT remain unclear. Considering that meta-analyses have revealed a dose–response association whereby an increase in RT frequency or volume produces larger increases in muscular fitness and hypertrophy (30, 31), these differences in RT frequency and the rate of participation in a long-term program could have contributed to the difference in the risk of incident hypertension between the Low-PA and High-PA groups. Moreover, a recent meta-analysis indicated that an RT frequency of three times a week or more produced greater BP-lowering effects compared with an RT frequency of two times a week (27). The mechanism of action of RT in BP has not been elucidated. Low-intensity RT decreased BP by enhancing endothelial NO-mediated vasodilatory function without concomitant increases in plasma norepinephrine levels, whereas high-intensity RT and concurrent Valsalva maneuver are likely to increase arterial stiffness by chronically increasing sympathetic activity and BP during exercise (32).

Nonetheless, although the risk of incident hypertension was significantly lower in women in the High-PA+RT group than in those in the High-PA group, the difference was not significant in men. To the best of our knowledge, only a few studies have examined sex-based differences in the antihypertensive effects of RT. A recent meta-analysis showed that RT significantly reduced SBP and DBP in women, but did not improve BP in men (27). According to an RCT, women exhibited a significant DBP-lowering effect without a concomitant increase in arterial stiffness after 4 weeks of moderate-intensity RT, whereas the RT program produced an increase in arterial stiffness in men (33). In another study, after 16 weeks of moderate-intensity RT, a significant reduction in arterial stiffness was observed in women but not in men (34). These results were consistent with the findings of the present study. Taken together, based on the above mentioned studies, potential sex differences in RT-related changes in arterial stiffness are likely due to sex-based differences in the risk of incident hypertension in the High-PA+RT group. However, training intensity, which was not considered in the present study, possibly plays an important role in the vascular benefits of RT. Therefore, additional RCTs are needed to investigate sex differences in RT-related antihypertensive effects by simultaneously considering both sex-specific effects and training intensity. Further longitudinal studies that consider long-term changes in arterial stiffness and BP are required to verify whether sex differences in RT-related changes in arterial stiffness could explain the difference in the risk of incident hypertension.

The major strength of our study is the long-term follow-up of up to 12 years in a prospective cohort of Korean adults. Moreover, we considered crucial confounders, including baseline BP, BMI, leisure-time PA levels, and diabetes mellitus, which are directly related to the risk for hypertension but were not considered in previous research. However, the present study had several limitations. First, there could be a recall bias because information on RT regularity and leisure-time PA levels was collected using self-reported questionnaires. Second, detailed information on RT intensity was unavailable for the present cohort. Therefore, we could not investigate the association between the RT intensity and the risk of incident hypertension. Further studies are needed to verify the optimal frequency, intensity, and training period to prevent hypertension. Finally, we only included Korean participants; thus, our findings may not be applicable to other populations.

In conclusion, our findings show that compliance with the leisure-time PA guidelines (≥150 min/week of moderate-intensity PA) may have preventive advantages against hypertension, whereas adding on RT to high levels of PA seems to be relatively more effective in preventing hypertension. In the subgroup analysis to examine whether there was an additive effect of RT on hypertension in the High-PA group, an additional reduction as observed only in women. However, as the training intensity, which was not considered in the present study, plays an important role in explaining the vascular benefits of RT, further studies are required to investigate sex differences in RT-related antihypertensive effects by simultaneously considering both sex and training intensity.

## Data availability statement

Publicly available datasets were analyzed in this study. This data can be found here: the Korean Genome and Epidemiology Study (KoGES; 4851-302), Korea National Institute of Health, Korea Disease Control and Prevention Agency.

## Ethics statement

The studies involving human participants were reviewed and approved by the Institutional Review Board Committee of the Korea National Institute of Health, Korea Disease Control and Prevention Agency (Approval No. 2021-04-02-P-A). The patients/participants provided their written informed consent to participate in this study.

## Author contributions

JHP and H-YP: conceptualization and writing—review and editing. H-YP and N-KL: methodology and resources. N-KL: software and data curation. JHP and N-KL: validation and formal analysis. JHP: investigation, writing—original draft preparation, and visualization. H-YP: supervision, project administration, and funding acquisition. All authors read and agreed to the published version of the manuscript.

## References

[1] Rapsomaniki E Timmis A George J Pujades-Rodriguez M Shah AD Denaxas S Blood pressure and incidence of twelve cardiovascular diseases: lifetime risks, healthy life-years lost, and age-specific associations in 1.25 million people. *Lancet.* (2014) 383:1899–911. 10.1016/S0140-6736(14)60685-1 24881994 PMC4042017

[2] Zhou D Xi B Zhao M Wang L Veeranki SP. Uncontrolled hypertension increases risk of all-cause and cardiovascular disease mortality in US adults: the NHANES III linked mortality study. *Sci Rep.* (2018) 8:9418. 10.1038/s41598-018-27377-2 29925884 PMC6010458

[3] NCD Risk Factor Collaboration [NCD-RisC]. Worldwide trends in hypertension prevalence and progress in treatment and control from 1990 to 2019: a pooled analysis of 1201 population-representative studies with 104 million participants. *Lancet.* (2021) 398:957–80. 10.1016/S0140-6736(21)01330-1 34450083 PMC8446938

[4] World Health Organization. *Global Recommendations on Physical Activity for Health.* Geneva: World Health Organization (2010). p. 8. 26180873

[5] Chase NL Sui X Lee DC Blair SN. The association of cardiorespiratory fitness and physical activity with incidence of hypertension in men. *Am J Hypertens.* (2009) 22:417–24. 10.1038/ajh.2009.6 19197248

[6] Mielke GI Bailey TG Burton NW Brown WJ. Participation in sports/recreational activities and incidence of hypertension, diabetes, and obesity in adults. *Scand J Med Sci Sports.* (2020) 30:2390–8. 10.1111/sms.13795 32757327

[7] Dimeo F Pagonas N Seibert F Arndt R Zidek W Westhoff TH. Aerobic exercise reduces blood pressure in resistant hypertension. *Hypertension.* (2012) 60:653–8. 10.1161/HYPERTENSIONAHA.112.197780 22802220

[8] Lopes S Mesquita-Bastos J Garcia C Bertoquini S Ribau V Teixeira M Effect of exercise training on ambulatory blood pressure among patients with resistant hypertension: a randomized clinical trial. *JAMA Cardiol.* (2021) 6:1317–23. 10.1001/jamacardio.2021.2735 34347008 PMC8340008

[9] Cornelissen VA Fagard RH Coeckelberghs E Vanhees L. Impact of resistance training on blood pressure and other cardiovascular risk factors: a meta-analysis of randomized, controlled trials. *Hypertension.* (2011) 58:950–8. 10.1161/HYPERTENSIONAHA.111.177071 21896934

[10] Garber CE Blissmer B Deschenes MR Franklin BA Lamonte MJ Lee IM American college of sports medicine position stand. Quantity and quality of exercise for developing and maintaining cardiorespiratory, musculoskeletal, and neuromotor fitness in apparently healthy adults: guidance for prescribing exercise. *Med Sci Sports Exerc.* (2011) 43:1334–59. 10.1249/MSS.0b013e318213fefb 21694556

[11] Piercy KL Troiano RP Ballard RM Carlson SA Fulton JE Galuska DA The physical activity guidelines for Americans. *JAMA.* (2018) 320:2020–8. 10.1001/jama.2018.14854 30418471 PMC9582631

[12] Whelton PK Carey RM Aronow WS Casey DE Jr Collins KJ Dennison Himmelfarb C 2017 ACC/AHA/AAPA/ABC/ACPM/AGS/APhA/ASH/ASPC/NMA/PCNA guideline for the prevention, detection, evaluation, and management of high blood pressure in adults: a report of the American college of cardiology/American heart association task force on clinical practice guidelines. *Hypertension.* (2018) 71:1269–324. 10.1161/HYP.0000000000000066 29133354

[13] Boeno FP Ramis TR Munhoz SV Farinha JB Moritz CEJ Leal-Menezes R Effect of aerobic and resistance exercise training on inflammation, endothelial function and ambulatory blood pressure in middle-aged hypertensive patients. *J Hypertens.* (2020) 38:2501–9. 10.1097/HJH.0000000000002581 32694343

[14] Polito MD Papst R Goessler K. Twelve weeks of resistance training performed with different number of sets: effects on maximal strength and resting blood pressure of individuals with hypertension. *Clin Exp Hypertens.* (2021) 43:164–8. 10.1080/10641963.2020.1833024 33043697

[15] Pagonas N Vlatsas S Bauer F Seibert FS Zidek W Babel N Aerobic versus isometric handgrip exercise in hypertension: a randomized controlled trial. *J Hypertens.* (2017) 35:2199–206. 10.1097/HJH.0000000000001445 28622156

[16] Park JH Jae SY. Resistance training does not mitigate cardiovascular reactivity to sympathoexcitation in young adults. *Exerc Sci.* (2019) 28:388–95. 10.15857/ksep.2019.28.4.388

[17] Kim Y Han BG KoGES group. Cohort profile: the Korean genome and epidemiology study (KoGES) consortium. *Int J Epidemiol.* (2017) 46:e20. 10.1093/ije/dyv316 27085081 PMC5837648

[18] Chobanian AV Bakris GL Black HR Cushman WC Green LA Izzo JL Jr. Seventh report of the joint national committee on prevention, detection, evaluation, and treatment of high blood pressure. *Hypertension.* (2003) 42:1206–52. 10.1161/01.HYP.0000107251.49515.c2 14656957

[19] Levey AS Coresh J Greene T Stevens LA Zhang YL Hendriksen S Using standardized serum creatinine values in the modification of diet in renal disease study equation for estimating glomerular filtration rate. *Ann Intern Med.* (2006) 145:247–54. 10.7326/0003-4819-145-4-200608150-00004 16908915

[20] Ishikawa-Takata K Tanaka H Nanbu K Ohta T. Beneficial effect of physical activity on blood pressure and blood glucose among Japanese male workers. *Diabetes Res Clin Pract.* (2010) 87:394–400. 10.1016/j.diabres.2009.06.030 19879663

[21] Williams PT. A cohort study of incident hypertension in relation to changes in vigorous physical activity in men and women. *J Hypertens.* (2008) 26:1085–93. 10.1097/HJH.0b013e3282fb81dc 18475145 PMC2828465

[22] Liu X Zhang D Liu Y Sun X Han C Wang B Dose-response association between physical activity and incident hypertension: a systematic review and meta-analysis of cohort studies. *Hypertension.* (2017) 69:813–20. 10.1161/HYPERTENSIONAHA.116.08994 28348016

[23] Korsager Larsen M Matchkov VV. Hypertension and physical exercise: the role of oxidative stress. *Medicina (Kaunas).* (2016) 52:19–27. 10.1016/j.medici.2016.01.005 26987496

[24] Liang J Zhang X Xia W Tong X Qiu Y Qiu Y Promotion of aerobic exercise induced angiogenesis is associated with decline in blood pressure in hypertension: result of excavation-CHN1. *Hypertension.* (2021) 77:1141–53. 10.1161/HYPERTENSIONAHA.120.16107 33611934

[25] Park JH Lim NK Park HY. Relative handgrip strength is inversely associated with hypertension in consideration of visceral adipose dysfunction: a nationwide cross-sectional study in Korea. *Front Physiol.* (2022) 13:930922. 10.3389/fphys.2022.930922 35928568 PMC9344337

[26] Abrahin O Moraes-Ferreira R Cortinhas-Alves EA Guerreiro JF. Is resistance training alone an antihypertensive therapy? A meta-analysis. *J Hum Hypertens.* (2021) 35:769–75. 10.1038/s41371-021-00582-9 34321596

[27] Oliver-Martínez PA Ramos-Campo DJ Martínez-Aranda LM Martínez-Rodríguez A Rubio-Arias JÁ. Chronic effects and optimal dosage of strength training on SBP and DBP: a systematic review with meta-analysis. *J Hypertens.* (2020) 38:1909–18. 10.1097/HJH.0000000000002459 32890263

[28] Cortez-Cooper MY Anton MM Devan AE Neidre DB Cook JN Tanaka H. The effects of strength training on central arterial compliance in middle-aged and older adults. *Eur J Cardiovasc Prev Rehabil.* (2008) 15:149–55. 10.1097/HJR.0b013e3282f02fe2 18391640

[29] Rossow LM Fahs CA Thiebaud RS Loenneke JP Kim D Mouser JG Arterial stiffness and blood flow adaptations following eight weeks of resistance exercise training in young and older women. *Exp Gerontol.* (2014) 53:48–56. 10.1016/j.exger.2014.02.010 24566193

[30] Grgic J Schoenfeld BJ Davies TB Lazinica B Krieger JW Pedisic Z. Effect of resistance training frequency on gains in muscular strength: a systematic review and meta-analysis. *Sports Med.* (2018) 48:1207–20. 10.1007/s40279-018-0872-x 29470825

[31] Schoenfeld BJ Ogborn D Krieger JW. Dose-response relationship between weekly resistance training volume and increases in muscle mass: a systematic review and meta-analysis. *J Sports Sci.* (2017) 35:1073–82. 10.1080/02640414.2016.1210197 27433992

[32] Figueroa A Okamoto T Jaime SJ Fahs CA. Impact of high- and low-intensity resistance training on arterial stiffness and blood pressure in adults across the lifespan: a review. *Pflugers Arch.* (2019) 471:467–78. 10.1007/s00424-018-2235-8 30426247

[33] Collier SR Frechette V Sandberg K Schafer P Ji H Smulyan H Sex differences in resting hemodynamics and arterial stiffness following 4 weeks of resistance versus aerobic exercise training in individuals with pre-hypertension to stage 1 hypertension. *Biol Sex Differ.* (2011) 2:9. 10.1186/2042-6410-2-9 21867499 PMC3184039

[34] Williams AD Ahuja KD Almond JB Robertson IK Ball MJ. Progressive resistance training might improve vascular function in older women but not in older men. *J Sci Med Sport.* (2013) 16:76–81. 10.1016/j.jsams.2012.05.001 22695137

