# Supplementary figures and images for: Association of leisure-time physical activity and resistance training with risk of incident hypertension: The Ansan and Ansung study of the Korean Genome and Epidemiology Study (KoGES)
Source: Front Cardiovasc Med. 2023 Jan 27;10:1068852. doi: 10.3389/fcvm.2023.1068852 (PMC9912934; doi:10.3389/fcvm.2023.1068852)

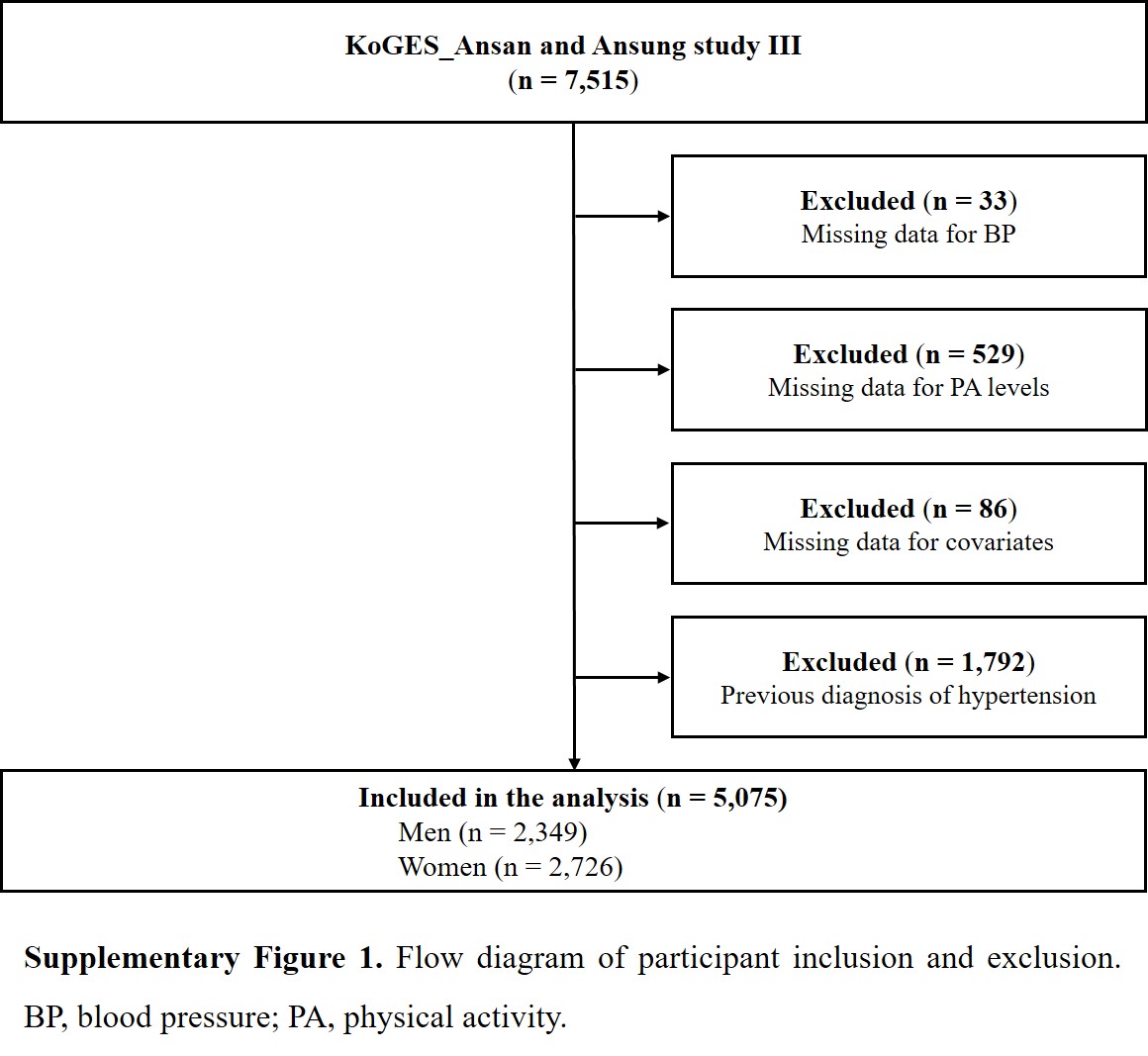

Supplement: Supplementary file 1 [file Image_1.jpg]
