# Supplementary material for: Association of leisure-time physical activity and resistance training with risk of incident hypertension: The Ansan and Ansung study of the Korean Genome and Epidemiology Study (KoGES)
Source: Front Cardiovasc Med. 2023 Jan 27;10:1068852. doi: 10.3389/fcvm.2023.1068852 (PMC9912934; doi:10.3389/fcvm.2023.1068852)
Supplement: Supplementary file 2 [file Table_1.docx]

**Supplementary Table 1.** Baseline characteristics of the participants stratified by sex and incident hypertension during the follow-up period

| **Variables** | **Men** (n = 2,349) | | ***p*-value** | **Women** (n = 2,726) | | ***p*-value** |
| --- | --- | --- | --- | --- | --- | --- |
|  | **Normotensive**  (n = 1,171) | **Hypertension**  (n = 1,178) |  | **Normotensive**  (n = 1,360) | **Hypertension**  (n = 1,366) |  |
| **Age** (years) | 53.69 ± 8.13 | 56.70 ± 8.71 | <0.0001 | 52.04 ± 7.29 | 58.70 ± 8.76 | <0.0001 |
| **Education level**, n (%) |  |  | <0.0001 |  |  | <0.0001 |
| ≤Elementary school | 172 (14.69) | 270 (22.92) ^a^ |  | 371 (27.28) | 759 (55.56) ^a^ |  |
| Middle/high school | 775 (66.18) | 763 (64.77) ^b^ |  | 905 (66.54) | 567 (41.51) ^b^ |  |
| ≥College | 224 (19.13) | 145 (12.31) ^c^ |  | 84 (6.18) | 40 (2.93) ^b^ |  |
| **Drinking habit**, n (%) |  |  | 0.06 |  |  | <0.01 |
| Never drinker | 260 (22.20) | 220 (18.68) |  | 946 (69.56) | 1,023 (74.89) ^a^ |  |
| Ex-drinker | 97 (8.29) | 118 (10.02) |  | 18 (1.32) | 23 (1.68) ^a,b^ |  |
| Current drinker | 814 (69.51) | 840 (71.31) |  | 396 (29.12) | 320 (23.43) ^b^ |  |
| **Smoking habit**, n (%) |  |  | 0.21 |  |  | 0.96 |
| Never smoker | 299 (25.53) | 288 (24.45) |  | 1,321 (97.13) | 1,329 (97.29) |  |
| Ex-smoker | 451 (38.52) | 425 (36.08) |  | 8 (0.59) | 8 (0.59) |  |
| Current smoker | 421 (35.95) | 465 (39.47) |  | 31 (2.28) | 29 (2.12) |  |
| **PA-time** (min/week) | 148.94 ± 175.46 | 122.25 ± 157.93 | <0.001 | 134.27 ± 155.08 | 105.11 ± 142.65 | <0.0001 |
| **RT**, n (%) | 167 (14.26) | 115 (9.76) | <0.001 | 148 (10.88) | 74 (5.42) | <0.0001 |
| **BMI** (kg/m^2^) | 23.43 ± 2.72 | 24.35 ± 2.86 | <0.0001 | 23.85 ± 2.76 | 25.19 ± 3.22 | <0.0001 |
| **WC** (cm) | 82.39 ± 7.20 | 86.19 ± 7.75 | <0.0001 | 79.62 ± 8.42 | 86.40 ± 9.27 | <0.0001 |
| **SBP** (mmHg) | 106.75 ± 9.59 | 117.15 ± 10.40 | <0.0001 | 103.24 ± 10.40 | 117.64 ± 10.76 | <0.0001 |
| **DBP** (mmHg) | 72.78 ± 6.93 | 78.70 ± 6.74 | <0.0001 | 69.47 ± 7.38 | 77.33 ± 7.19 | <0.0001 |
| **T-Chol** (mg/dL) | 185.99 ± 32.54 | 186.68 ± 33.98 | 0.61 | 192.05 ± 33.31 | 195.73 ± 34.55 | <0.01 |
| **HDL-C** (mg/dL) | 43.29 ± 10.37 | 42.10 ± 10.04 | <0.01 | 46.87 ± 10.15 | 44.32 ± 9.76 | <0.0001 |
| **TG** (mg/dL) | 135.12 ± 95.15 | 156.73 ± 125.75 | <0.0001 | 111.11 ± 62.17 | 136.61 ± 85.26 | <0.0001 |
| **FBG** (mg/dL) | 92.10 ± 13.21 | 94.91 ± 15.70 | <0.0001 | 87.47 ± 10.56 | 91.38 ± 13.59 | <0.0001 |
| **Creatinine** (mg/dL) | 1.07 ± 0.12 | 1.06 ± 0.14 | 0.69 | 0.87 ± 0.09 | 0.89 ± 0.22 | <0.01 |
| **eGFR**  (mL/min per 1.73 m^2^) | 73.78 ± 10.51 | 73.37 ± 10.70 | 0.35 | 69.40 ± 8.52 | 66.80 ± 9.60 | <0.0001 |
| **DM**, n (%) | 93 (7.94) | 156 (13.24) | <0.0001 | 66 (4.85) | 172 (12.59) | <0.0001 |

PA**-**time, total time spent participating regularly in any sports or exercise to the point of sweating; BMI, body mass index; WC, waist circumference; SBP, systolic blood pressure; DBP, diastolic blood pressure; T-Chol, total cholesterol; HDL-C, high-density lipoprotein cholesterol; TG, triglycerides; FBG, fasting blood glucose; eGFR, estimated glomerular filtration rate; DM, diabetes mellitus; Categories marked with the same letter are not significantly different by post-hoc pairwise comparisons.
