# Supplementary material for: Association of leisure-time physical activity and resistance training with risk of incident hypertension: The Ansan and Ansung study of the Korean Genome and Epidemiology Study (KoGES)
Source: Front Cardiovasc Med. 2023 Jan 27;10:1068852. doi: 10.3389/fcvm.2023.1068852 (PMC9912934; doi:10.3389/fcvm.2023.1068852)
Supplement: Supplementary file 3 [file Table_2.docx]

**Supplementary Table 2.** Baseline characteristics of participants, stratified based on leisure-time PA levels and RT regularity

| **Variables** | **Low-PA** (n = 3,145) | | ***p*-value** | **High-PA** (n = 1,930) | | ***p*-value** |
| --- | --- | --- | --- | --- | --- | --- |
|  | **Low-PA**  (n = 3,052) | **Low-PA+RT**  (n = 93) |  | **High-PA**  (n = 1,519) | **High-PA+RT**  (n = 411) |  |
| **Age** (years) | 56.75 ± 8.90 | 51.47 ± 7.57 | <0.0001 | 53.95 ± 7.96 | 50.35 ± 6.13 | <0.0001 |
| **Women**, n (%) | 1,707 (55.93) | 37 (39.78) | <0.01 | 797 (52.47) | 185 (45.01) | <0.01 |
| **Education level**, n (%) |  |  | <0.0001 |  |  | <0.0001 |
| ≤Elementary school | 1,210 (39.65) | 9 (9.68) ^a^ |  | 319 (21.00) | 34 (8.27) ^a^ |  |
| Middle/high school | 1,642 (53.80) | 73 (78.49) ^b^ |  | 1,004 (66.10) | 291 (70.80) ^b^ |  |
| ≥College | 200 (6.55) | 11 (11.83) ^b^ |  | 196 (12.90) | 86 (20.93) ^c^ |  |
| **Drinking habit**, n (%) |  |  | <0.05 |  |  | 0.06 |
| Never drinker | 1,557 (51.01) | 37 (39.78) ^a^ |  | 693 (45.62) | 162 (39.42) |  |
| Ex-drinker | 151 (4.95) | 9 (9.68) ^b^ |  | 77 (5.07) | 19 (4.62) |  |
| Current drinker | 1,344 (44.04) | 47 (50.54) ^a,b^ |  | 749 (49.31) | 230 (55.96) |  |
| **Smoking habit**, n (%) |  |  | <0.01 |  |  | 0.19 |
| Never smoker | 1,981 (64.91) | 50 (53.76) ^a^ |  | 964 (63.46) | 242 (58.88) |  |
| Ex-smoker | 458 (15.01) | 25 (26.88) ^b^ |  | 310 (20.41) | 99 (24.09) |  |
| Current smoker | 613 (20.08) | 18 (19.36) ^a^ |  | 245 (16.13) | 70 (17.03) |  |
| **PA-time** (min/week) | 34.69 ± 50.94 | 90.74 ± 43.11 | <0.0001 | 312.30 ± 158.39 | 379.28 ± 178.36 | <0.0001 |
| **BMI** (kg/m^2^) | 24.10 ± 3.12 | 24.64 ± 2.93 | 0.10 | 24.40 ± 2.78 | 24.46 ± 2.57 | 0.65 |
| **WC** (cm) | 84.32 ± 8.94 | 84.43 ± 9.17 | 0.91 | 82.75 ± 8.15 | 81.33 ± 8.49 | <0.01 |
| **SBP** (mmHg) | 112.27 ± 12.27 | 110.29 ± 10.25 | 0.07 | 109.78 ± 11.84 | 108.16 ± 11.63 | <0.05 |
| **DBP** (mmHg) | 75.07 ± 7.93 | 74.63 ± 7.69 | 0.60 | 73.80 ± 7.99 | 72.69 ± 8.04 | <0.05 |
| **T-Chol** (mg/dL) | 188.79 ± 34.22 | 190.25 ± 33.78 | 0.68 | 193.24 ± 33.23 | 191.89 ± 32.84 | 0.46 |
| **HDL-C** (mg/dL) | 43.83 ± 10.04 | 44.05 ± 9.75 | 0.83 | 44.51 ± 10.33 | 46.50 ± 10.96 | <0.001 |
| **TG** (mg/dL) | 135.86 ± 100.85 | 144.63 ± 117.58 | 0.48 | 133.84 ± 86.40 | 119.65 ± 69.27 | <0.001 |
| **FBG** (mg/dL) | 91.21 ± 13.02 | 93.91 ± 17.09 | 0.15 | 91.20 ± 14.46 | 91.04 ± 12.61 | 0.83 |
| **Creatinine** (mg/dL) | 0.95 ± 0.15 | 1.00 ± 0.15 | <0.01 | 0.98 ± 0.23 | 1.01 ± 0.14 | <0.01 |
| **eGFR**  (mL/min per 1.73 m^2^) | 71.00 ± 10.61 | 72.35 ± 13.34 | 0.34 | 70.07 ± 9.67 | 69.61 ± 8.21 | 0.33 |
| **DM**, n (%) | 287 (9.40) | 11 (11.83) | 0.43 | 153 (10.07) | 36 (8.76) | 0.43 |

PA, physical activity; RT, resistance training; PA**-**time, total time spent for participating regularly in any sport or exercise to the point of sweating; min, minute; BMI, body mass index; WC, waist circumference; SBP, systolic blood pressure; DBP, diastolic blood pressure; T-Chol, total cholesterol; HDL-C, high-density lipoprotein cholesterol; TG, triglycerides; FBG, fasting blood glucose; DM, diabetes mellitus; Categories marked with the same letter are not significantly different by post-hoc pairwise comparisons.
