# Supplementary material for: Association of leisure-time physical activity and resistance training with risk of incident hypertension: The Ansan and Ansung study of the Korean Genome and Epidemiology Study (KoGES)
Source: Front Cardiovasc Med. 2023 Jan 27;10:1068852. doi: 10.3389/fcvm.2023.1068852 (PMC9912934; doi:10.3389/fcvm.2023.1068852)
Supplement: Supplementary file 4 [file Table_3.docx]

**Supplementary Table 3.** Hazard ratios for new-onset hypertension according to RT regularity and sex in participants with high leisure-time PA levels

|  | **N** | **Total person-years** | **Participants with hypertension**, n (%) | **Event rate** (1,000-person year) | **PA-time** (min/week) | **RT Levels** | | | **Crude Model,**  **HR** (95% CI) | **Adjusted Model,**  **HR** (95% CI) |
| --- | --- | --- | --- | --- | --- | --- | --- | --- | --- | --- |
|  |  |  |  |  |  | **Frequency** | **Training Period** | |  |  |
|  |  |  |  |  |  | (days/week) | (month) | ≥1 year (%) |  |  |
| **Total** |  |  |  |  |  |  |  |  |  |  |
| High-PA | 1,519 | 12,358.07 | 629 (41.41) | 50.90 | 312.30 ± 158.39 | - | - | - | 1 (reference) | 1 (reference) |
| High-PA+RT | 411 | 3,644.72 | 139 (33.82) | 38.14 | 379.28 ± 178.36 | 4.69 ± 1.48 | 67.12 ± 81.23 | 74.21 | 0.77 (0.64–0.94) ^*^ | 0.80 (0.66–0.99) ^*^ |
| **Men** |  |  |  |  |  |  |  |  |  |  |
| High-PA | 722 | 5,747.93 | 307 (42.52) | 53.41 | 321.62 ± 162.71 | - | - | - | 1 (reference) | 1 (reference) |
| High-PA+RT | 226 | 1,911.49 | 83 (36.73) | 43.42 | 376.58 ± 188.96 | 4.66 ± 1.62 | 85.47 ± 96.78 ^a^ | 77.88 | 0.83 (0.64–1.07) | 0.89 (0.68–1.17) |
| **Women** |  |  |  |  |  |  |  |  |  |  |
| High-PA | 797 | 6,610.14 | 322 (40.40) | 48.71 | 303.86 ± 153.99 | - | - | - | 1 (reference) | 1 (reference) |
| High-PA+RT | 185 | 1,733.23 | 56 (30.27) | 32.31 | 382.59 ± 164.95 | 4.72 ± 1.32 | 45.80 ± 50.66 ^a^ | 69.73 | 0.68 (0.50–0.93) ^*^ | 0.65 (0.46–0.91) ^*^ |

RT, resistance training; PA, physical activity; PA**-**time, time spent participating regularly in any sports or exercise to the point of sweating; HR, hazard ratio; CI, confidence interval; BMI, body mass index; T-Chol, total cholesterol; SBP, systolic blood pressure; eGFR, estimated glomerular filtration rate; ^a^, *p*<0.05 compared female with male in High-PA+RT; ^*^, *p*<0.05; Adjusted for age, sex, drinking, smoking, education level, BMI, T-Chol, SBP, eGFR, diabetes mellitus, and PA time.
