# Supplementary material for: Association of leisure-time physical activity and resistance training with risk of incident hypertension: The Ansan and Ansung study of the Korean Genome and Epidemiology Study (KoGES)
Source: Front Cardiovasc Med. 2023 Jan 27;10:1068852. doi: 10.3389/fcvm.2023.1068852 (PMC9912934; doi:10.3389/fcvm.2023.1068852)
Supplement: Supplementary file 5 [file Table_4.docx]

**Supplementary Table 4.** Hazard ratios for new-onset hypertension according to leisure-time PA levels in various subgroups

| **Subgroups** | **N** | **Participants with hypertension,** n (%) | **Leisure-time PA levels** | | | ***p* for interaction** |
| --- | --- | --- | --- | --- | --- | --- |
|  |  |  | **Events** (event rate ^a^) | | **HR** (95% CI)  Low-PA vs. High-PA |  |
|  |  |  | **Low-PA** | **High-PA** |  |  |
| **Age** (years) |  |  |  |  |  |  |
| <55 | 2,751 | 1,050 (38.17) | 658 (54.01) | 392 (35.30) | 0.65 (0.57–0.75) ^****^ | 0.97 |
| ≥55 | 2,324 | 1,494 (64.29) | 1,118 (128.34) | 376 (76.76) | 0.69 (0.60–0.78) ^****^ |  |
| **Sex** |  |  |  |  |  |  |
| Male | 2,349 | 1,178 (50.15) | 788 (86.26) | 390 (50.92) | 0.70 (0.62–0.81) ^****^ | 0.18 |
| Female | 2,726 | 1,366 (50.11) | 988 (84.02) | 378 (45.31) | 0.66 (0.57–0.75) ^****^ |  |
| **BMI** (kg/m^2^) |  |  |  |  |  |  |
| <25 | 3,186 | 1,385 (43.47) | 984 (68.15) | 401 (38.55) | 0.74 (0.64–0.84) ^****^ | 0.06 |
| ≥25 | 1,889 | 1,159 (61.36) | 792 (122.67) | 367 (65.52) | 0.62 (0.54–0.71) ^****^ |  |
| **Current drinking habit** |  |  |  |  |  |  |
| No | 2,705 | 1,384 (51.16) | 1,019 (89.49) | 365 (45.55) | 0.62 (0.54–0.70) ^****^ | <0.01 |
| Yes | 2,370 | 1,160 (48.95) | 757 (79.61) | 403 (50.44) | 0.75 (0.66–0.86) ^****^ |  |
| **Smoking status** |  |  |  |  |  |  |
| Never | 4,129 | 2,050 (49.65) | 1,417 (84.01) | 633 (46.81) | 0.69 (0.62–0.76) ^****^ | 0.98 |
| Ever | 946 | 494 (52.22) | 359 (89.12) | 135 (54.43) | 0.65 (0.52–0.81) ^***^ |  |
| **Diabetes mellitus** |  |  |  |  |  |  |
| No | 4,588 | 2,216 (48.30) | 1,561 (80.18) | 655 (44.28) | 0.67 (0.61–0.74) ^****^ | 0.65 |
| Yes | 487 | 328 (67.35) | 215 (150.65) | 113 (93.40) | 0.70 (0.54–0.89) ^**^ |  |

PA, physical activity; HR, hazard ratio; CI, confidence interval; BMI, body mass index; T-Chol, total cholesterol; SBP, systolic blood pressure; eGFR, estimated glomerular filtration rate; ^a^, event rate is presented per 1,000-person year of follow-up; ^**^, *p*<0.01; ^***^, *p*<0.001; ^****^, *p*<0.0001; Adjusted for age, sex, drinking, smoking, education level, BMI, T-Chol, SBP, eGFR, and diabetes mellitus.
